# Efficacy, feasibility, and safety of aquablation after previous urolift treatment of lower urinary tract symptoms secondary to benign prostate hyperplasia: a retrospective cohort study

**DOI:** 10.1007/s00345-025-05897-7

**Published:** 2025-09-03

**Authors:** Hashem Darwazeh, Keng Lim Ng, Neil Barber

**Affiliations:** Urology Department – Frimley Park Hospital NHS Foundation Trust, Portsmouth Road, Frimley, Camberley, GU16 7UJ England

**Keywords:** Aquablation, Minimally invasive surgical therapies, Urolift, Benign prostate hyperplasia

## Abstract

**Purpose:**

The influence of a previous Urolift treatment on the outcomes of prostate Aquablation is still controversial. This retrospective cohort study aimed to evaluate the perioperative outcomes, efficacy, feasibility, and safety of Aquablation after previous Urolift treatment.

**Methods:**

The charts of patients with benign prostate hyperplasia (BPH) complicated by storage and voiding symptoms, who were previously treated with Urolift followed by Aquablation between January 2022 and July 2024, were retrospectively reviewed and analyzed for changes in International Prostate Symptom Score (IPSS), maximum urinary flow rates (Qmax), and postvoid residual volume (PVR) from baseline (pre-Aquablation) to the mean of three months postoperatively. Day-case surgery was performed in 75% of the cases.

**Results:**

The study included 40 patients with a mean age of 68 (SD ± 8.91) years with moderately enlarged prostates (mean volume 81.56 mL, (SD ± 25.32), and median PSA 3.2 ng/dL). Five patients were presented initially with an indwelling Foley catheter, so their data were not included in the statistical analysis. After Aquablation, the mean IPSS improved from 24.7 (SD ± 7.63) at baseline to 9.8 (SD ± 2.55) (*p* < 0.0001), the mean Qmax increased from 9.6mL/sec (SD ± 5.76) at baseline to 20.8mL/sec (SD ± 6.28) (*p* < 0.0001), and the mean PVR decreased from 143mL (SD ± 104.89) at baseline to 36mL (SD ± 30.63 ) (*p* < 0.0001). The hospital stay for patients admitted ranged from 1 to 2 days. Moreover, there were no major intraoperative difficulties removing dislodged Urolift clips with a loop resectoscope at the end of the procedure, while non-dislodged clips were left in situ. None of the patients had postoperative bleeding that required hospital admission or blood transfusion following discharge.

**Conclusion:**

Based on these results, prostate Aquablation appeared to be an effective, safe, feasible, and reliable surgical procedure for BPH patients who have had previous Urolift treatment. Further prospective and larger scale studies are needed.

## Introduction

Management of lower urinary tract symptoms (LUTS) secondary to benign prostatic hyperplasia (BPH) has evolved significantly with the introduction of minimally invasive surgical therapies (MIST), including Urolift, Rezum, intraprostatic stents, and iTIND [1]. These MIST options aim to reduce the burden of LUTS while minimizing the complications associated with traditional surgical procedures like transurethral resection of the prostate (TURP). Advantages of MIST include reduced perioperative morbidity, shorter recovery time, and preservation of sexual function; an increasingly important factor in patient decision making. However, their limitations include variable long-term durability and higher retreatment rates compared with TURP. Consequently, MIST procedures have become an appealing option for patients prioritising quicker recovery and the preservation of sexual health, even though long-term durability remains a relative limitation.

The outpatient procedure for treating LUTS caused by BPH, a prostatic urethral lift (PUL) or Urolift, can be performed under local anesthesia. The urethra is drawn under tension while multiple non-absorbable monofilament sutures are placed through the prostatic urethra to the lateral lobes [2]. Recent evidence, including a systematic review by Moretto et al., shows considerable variation in anaesthesia protocols and supports the feasibility of performing UroLift under local anaesthesia. However, it also highlights real-world limitations, particularly reduced durability in larger prostates, anatomical restrictions, and the possibility of retreatment in some patients [3]. According to the American, Canadian, and European Urological Association guidelines, most of these options are not recommended for prostates larger than 80mL [4–6]. With an expected increase in complications in larger prostates, Aquablation clinically improves outcomes in patients with 30-80mL prostates and 80-150mL prostates for LUTS/BPH [7]. This novel minimally invasive surgery for BPH, Aquablation, is performed by mechanically removing prostatic adenoma using a high-speed waterjet under surgical control and robotic execution. Using real-time intraprocedural ultrasound, the ergonomically designed waterjet cuts based on the contour plan specified by the surgeon, while preserving the bladder neck, verumontanum, peripheral sphincter, and neurovascular bundles during the procedure. Due to the robotic and heatless nature of Aquablation, it is more precise and faster than traditional heat-based approaches for removing prostatic adenoma [8]. There is still controversy regarding the impact of a prior Urolift treatment on the results of prostate Aquablation. Therefore, this retrospective cohort study was performed to assess the outcome of the Aquablation technique on a group of patients with BPH who were previously treated with Urolift by comparing the mean International Prostate Symptom Score (IPSS), maximum urinary flow rates (Qmax), and postvoid residual volume (PVR) pre- and postoperatively.

## Methods

### Study design and patient population

This retrospective cohort study was conducted at Frimley Park Hospital NHS Foundation Trust (Surrey, UK). The charts of patients with BPH complicated by storage and voiding symptoms, who were previously treated with Urolift followed by Aquablation, between January 2022 and July 2024, were retrospectively reviewed. The study included a total of 40 men diagnosed with moderate to severe LUTS secondary to BPH. All the patients had previously undergone prostatic urethral lifts (PULs) or Urolift prostate procedure (UroLift System, Neotract Inc, Teleflex, Pleasanton, CA) in a mean timeframe of 47 months, followed by Aquablation therapy. At our centre, specific criteria were used to determine suitability for same-day discharge (SDD). All patients underwent a preoperative anaesthesia assessment to evaluate overall fitness for day-case surgery. Exclusion criteria included an inability to stop anticoagulation, significant cardiac dysfunction (NYHA Class III/IV), poor functional status, or medical frailty. Additionally, patients treated in the private sector were not considered for SDD due to institutional policy, even if they met all clinical criteria. An evaluation was conducted on all men up to three months following their Aquablation surgery.

Given that this is a retrospective study, we recognize there were some important limitations. There may be a degree of selection bias, as the decision to proceed with Aquablation after Urolift was based on clinical judgment rather than a standardized protocol. This also means we had less control over potential confounding factors.

### Preoperative evaluation

Pertinent clinical data were collected on 35 patients (not initially presented with an indwelling Foley catheter) before and after Aquablation. IPSS questionnaire was utilized to assess the severity of symptoms. Qmax and PVR were measured prior to Aquablation in each patient. Flexible cystoscopy and transrectal ultrasound (TRUS) prostate volume measurements were performed in all patients as part of the preoperative workup to rule out urethral strictures, assess for intravesical pathology, and directly visualize the prostatic urethra and bladder neck anatomy. This step was especially important in those with prior MIST procedures such as Urolift, as it allowed precise confirmation of bladder outlet obstruction and helped guide surgical planning. Postoperative comparisons and outcome assessments were based on the above metrics.

### Surgical technique

**Fig. 1:**
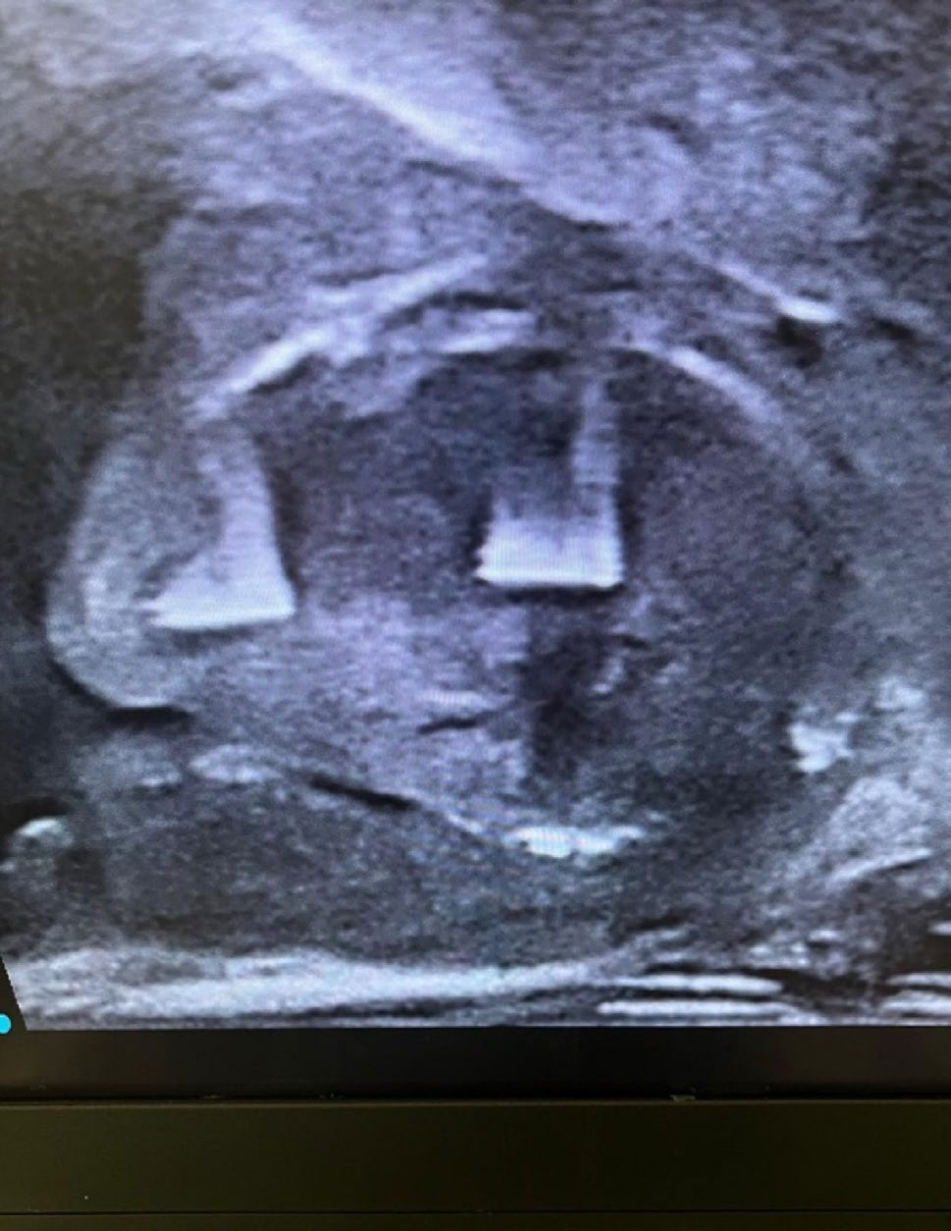
Transrectal ultrasound showing the prostatic Urolift clips prior to Aquablation

**Fig. 2:**
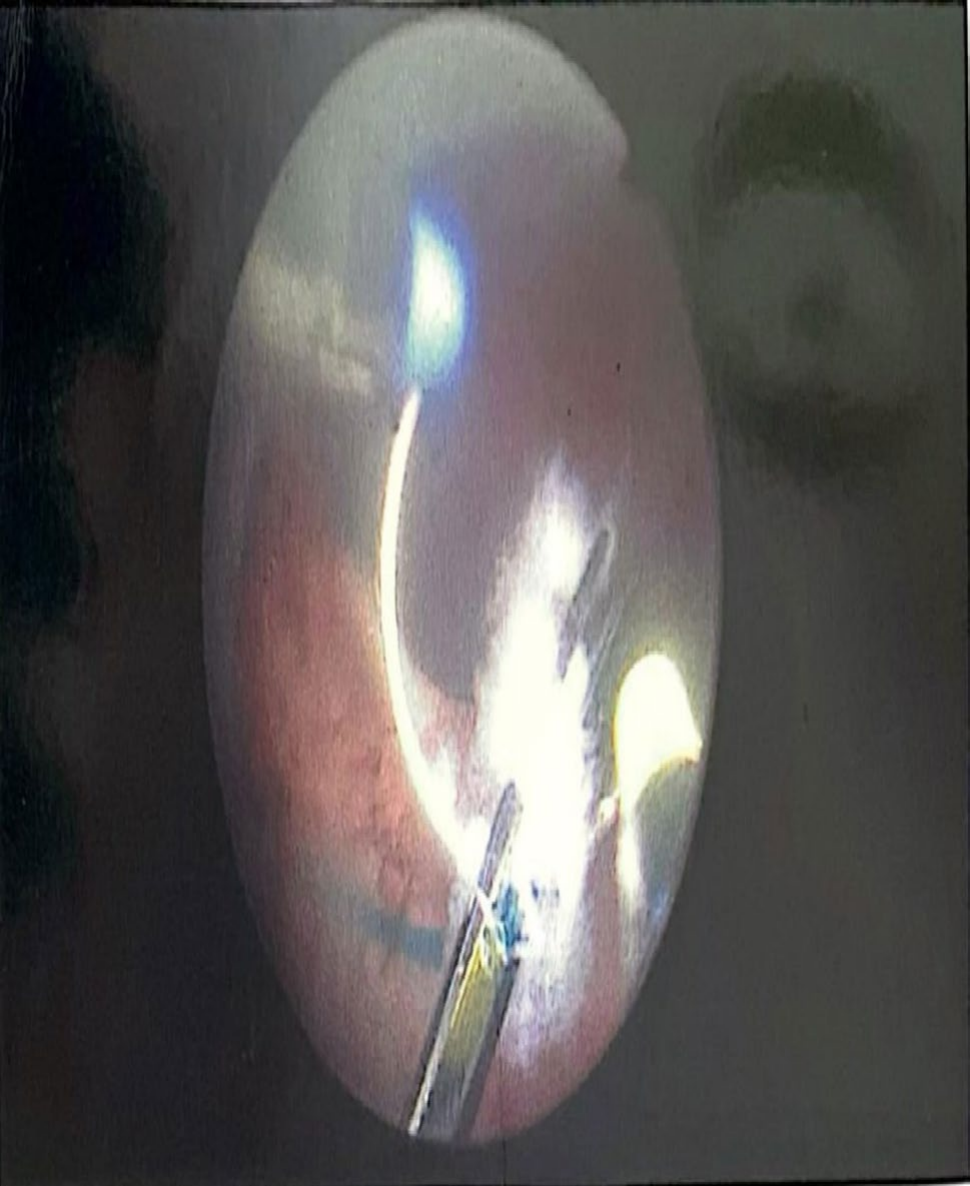
Bipolar plasma loop used for cutting through the suture line of the Urolift clip

**Fig. 3:**
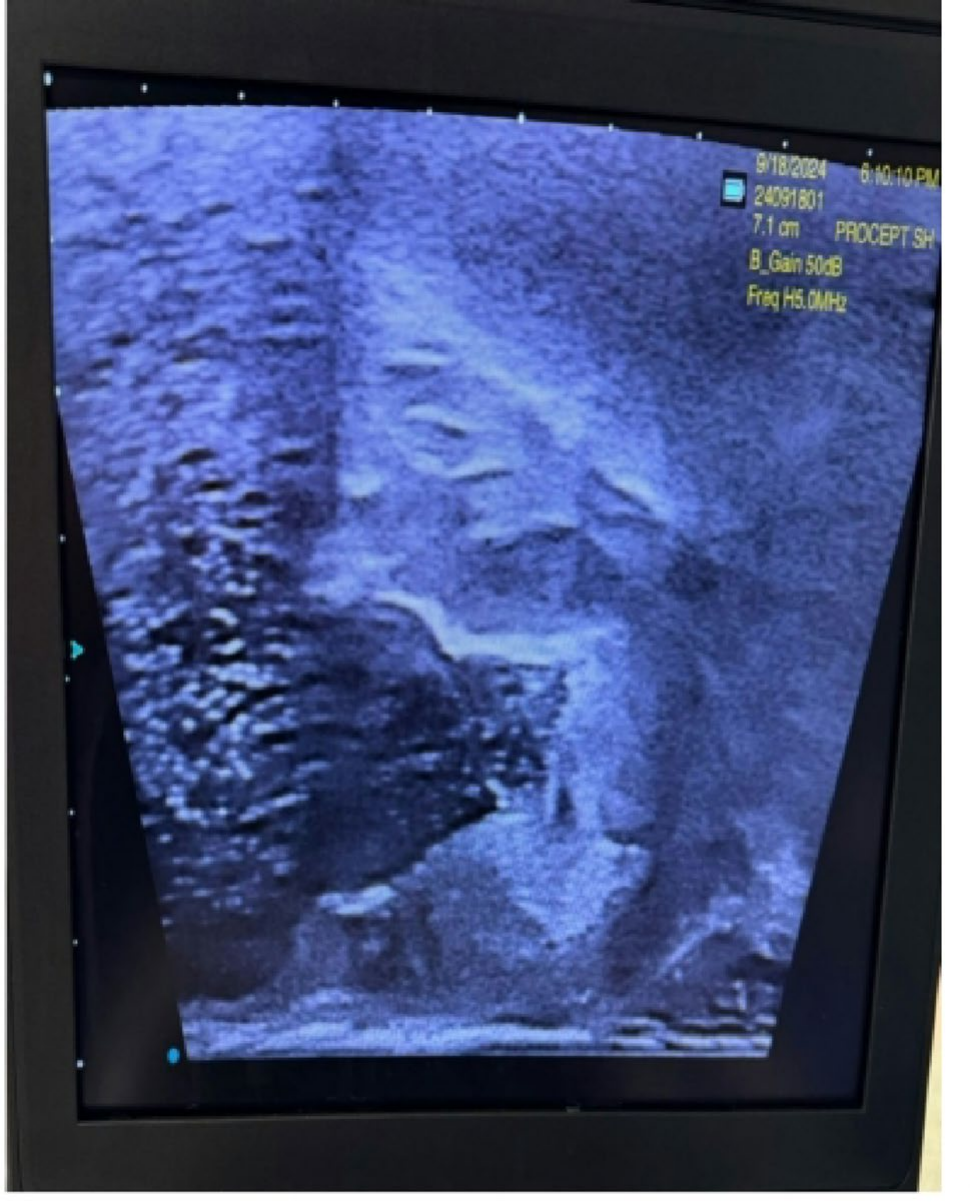
Transrectal ultrasound showing a wide prostatic cavity after Aquablation

Under general anaesthesia, TRUS was performed for all patients to identify the site and number of previously applied prostatic Urolift clips (Fig. 1). The AquaBeam Robotic System (AquaBeam System, PROCEPT BioRobotics Inc., USA) was used for Aquablation in two passes. Olympus Bipolar PlasmaLoop 26 Fr. was used during all procedures for targeted coagulation of the prostatic fossa, primarily at the bladder neck and resected areas prone to oozing, to avoid excessive thermal injury while achieving haemostasis. Where feasible, preservation of the bladder neck was prioritised, and selective resection of residual anterior bladder neck tissue was undertaken to achieve a smooth, wide prostatic cavity and prevent obstruction [9]. The exposed Urolift clips were removed using the bipolar plasma loop by cutting through the suture line along with adjacent prostatic tissue (Fig. 2). The remaining unexposed clips were left in-situ. All procedures were performed by surgeons experienced with both Urolift and Aquablation. While prior Urolift implants introduced an additional step, no significant learning curve or increase in operative time was observed in this cohort. A 22 Fr. silicone soft 3-way catheter was used for all patients at the end of the procedure, with 30-40mls of sterile water infused into the catheter balloon after confirmation of the favourable wide prostatic cavity on TRUS (Fig. 3).

### Postoperative assessment and patient discharge

The patients were closely monitored in the recovery room for 4–6 h following the procedure. Inflow bladder irrigation was stopped 1–2 h after the procedure, and catheter output colour was monitored. All patients with rosy-coloured urine were discharged the same day with follow-up instructions for de-catheterisation. An outpatient appointment with a new IPSS questionnaire and uroflowmetry was scheduled at a mean of three months after Aquablation.

The normality of differences between pre- and postoperative measurements was assessed using the Shapiro–Wilk test. As all variables (IPSS, Qmax, and PVR) met the normality assumption (*p* > 0.05), the paired t-test was applied.

## Results

### Patient demographics and aquablation outcomes

The average age of the study subjects was 68 years (range 47–83 years). The median prostate-specific antigen (PSA) level was 3.6ng/dL (range 0.52- 9.3ng/dL), and the mean prostate volume was 81.56mL (SD ± 25.32), ranging between 41 and 147mL as determined by TRUS. Five patients (12.5%) were primarily presented with urinary retention with an indwelling Foley catheter, for whom surgery was performed, but were omitted from the statistical analysis of pre- and post-operative data due to the lack of pre-operative data. The mean preoperative IPSS for the remaining 35 patients was 24.7 (SD ± 7.63), ranging between 15 and 33, reflecting the significant severity of urinary symptoms. The mean baseline Qmax was 9.6mL/sec (SD ± 5.76), ranging between 2.9 and 18mL/sec, and the mean PVR was 143mL ± 104.89, ranging between 55-350mL. The procedure demonstrated a meaningful improvement in urinary function, with the mean 3-month IPSS dropping to 9.8 ± 2.55, ranging between 6 and 14 (*p* < 0.0001). The mean Qmax increased notably to 20.8mL/sec ± 6.28, ranging between 12.3 and 34mL/sec (*p* < 0.0001), and the mean PVR volume dropped to 36mL ± 30.63 (range 2- 94mL, *p* < 0.0001).

It is important to note that five patients were excluded from the statistical analysis due to presenting with indwelling Foley catheters at baseline. While their exclusion was necessary due to a lack of preoperative IPSS, Qmax, and PVR data, this may introduce a degree of selection bias. These patients potentially represent a subgroup with more severe LUTS or advanced disease, which could limit the generalizability of our findings to all patients undergoing Aquablation after Urolift. Including such cases in future prospective studies may provide a more complete picture of outcomes across the full clinical spectrum.

### Early post-operative evaluation and complications

Thirty patients (75%) were discharged on the same surgery day following Aquablation, with a catheter removal time of three days, while ten patients (25%) were kept in the hospital following the surgery. Seven out of those ten patients had their surgery performed in the private sector. They were observed for 2 nights according to the private ward policy, despite not having any complications and were later discharged after a successful trial without a catheter. In the remaining three cases, two patients remained overnight (1 night) for social reasons at their preference and were discharged the following day with a three-day catheter removal time. Only one patient (2.5%) experienced an early postoperative complication, presenting with haematuria requiring overnight (1 night) observation and continuous bladder irrigation. This was classified as a Clavien–Dindo Grade I complication, as it resolved without the need for surgical, endoscopic, or radiological intervention and the catheter was removed after 5 days.

During the first 30 days following hospital discharge, only two emergency room visits out of the 40 patients (5%) were reported. In one case, a patient presented with haematuria and a blocked catheter, which was managed by manual clot irrigation (Clavien–Dindo Grade II), and the patient was then discharged. In the second case, a simple urinary tract infection was treated with selective oral antibiotics (Clavien–Dindo Grade II).

It is worth mentioning that no patients required blood transfusions, and there were no hospitalizations or returns to the operating room for bleeding or haemostasis.

## Discussion

BPH can initially be treated conservatively by watching and lifestyle modification, as well as with medications such as alpha blockers, 5-alpha reductase inhibitors, and more recently, phosphodiesterase inhibitors [6]. On the other hand, bothersome LUTS/BPH are generally treated surgically in non-responders or those who cannot tolerate the side effects associated with the medical treatment [10]. 

The development of several minimally invasive surgical therapies (MIST) for BPH in the last few years has provided patients with therapeutic options involving lower morbidity rates as alternatives to transurethral resection of the prostate (TURP), particularly the lower risk of negative impact upon sexual functions [6]. 

Several concerns have been raised regarding the durability of the newly launched MIST due to the relatively high rate of reoperations and/or continued medical therapy after surgery [11]. Previous studies have raised concerns about the long-term durability of newer MIST procedures, with higher rates of retreatment or ongoing medical therapy compared with TURP. For example, Roehrborn et al. found a 13.6% retreatment rate at 5 years after Urolift, largely due to regrowth of adenomatous tissue beyond prostatic implants [12]. By contrast, Aquablation has shown sustained symptom relief with minimal impact on sexual function, even in larger prostates, as demonstrated in the WATER and WATER II trials [7]. Therefore, mounting numbers of patients are being seen who were previously treated effectively with Urolift and are subsequently experiencing symptoms of prostatic adenoma regrowth beyond the lift of implants. The same patients who most often have chosen MIST to protect sexual functions are likely to still have the same desires if further surgical intervention is needed or offered.

In this study, we performed Aquablation after Urolift for deteriorating cases of LUTS with confirmed bladder outlet obstruction due to BPH on flexible cystoscopy. The benefits of Aquablation are evident in the fact that it leaves a large prostate cavity, which leads to good flow outcomes. Due to the non-thermal technique as well as the unique apical ablation through surgical mapping, Aquablation offers patients the benefit of a very low risk of erectile dysfunction at around 0% and a minimal risk of dry ejaculation at 10.8% [13]. 

In the first three months after Aquablation, the average IPSS was reduced by 60%, the mean Qmax was elevated by 54%, and the mean PVR was reduced by 75% as compared to baseline, which indicates a significant improvement in urinary symptoms. When compared to published trial data, our results align closely with Aquablation outcomes in patients without prior Urolift. In the WATER trial, for example, the mean IPSS improved by about 16 points and Qmax increased by roughly 10 mL/sec at three months in prostates between 30 and 80 mL. Similarly, in WATER II, which included larger prostates (80–150 mL), improvements remained significant [7]. Our study showed a mean IPSS improvement of 14.9 points and a Qmax increase of 11.2 mL/sec, despite the added factor of previous Urolift implants. This suggests that Aquablation remains equally effective even as a secondary intervention. From a theoretical standpoint, prior Urolift implantation could potentially present challenges to subsequent Aquablation. For example, retained UroLift clips may potentially interfere with anatomical orientation, disrupt tissue contour mapping, or alter the trajectory of the waterjet during the procedure. However, because Aquablation is guided by real-time ultrasound and robotic precision, these risks appear minimised. Notably, one series reported that Aquablation remains safe and effective even after prior treatments, including Urolift, without increasing the risk of tissue injury or bleeding [14]. Since no significant postoperative complications were reported (primarily limited to minor events (Clavien–Dindo Grades I–II)), and the rate of emergency room visits was remarkably low (5%), the present results support the safety of the procedure. Our initial early experience of Aquablation after Urolift is the first to report on its feasibility. This procedure was performed in all patients with the standard treatment planning and post-ablation diathermy/resection, with no difficulties encountered in removing the dislodged implants or a significant increase in operative time. In addition, the postoperative care was carried out according to the standard Aquablation management protocols. This cohort study has clearly demonstrated that Aquablation is a feasible procedure, does not involve significant complications, and has good postoperative outcomes.

We acknowledge that the relatively small sample size (*n* = 35) limits the statistical power of this study and may affect the external validity of our findings. A formal power analysis was not conducted prior to data collection due to the retrospective nature of the study and the focus on evaluating outcomes within a specific, real-life patient cohort. Nevertheless, the statistically significant improvements observed across key clinical parameters suggest that the sample size was sufficient to detect meaningful trends. Future prospective studies with larger, more diverse populations are needed to validate these findings and assess long-term outcomes more comprehensively.

## Conclusion

Aquablation appears to be a safe, feasible, and effective treatment option for patients with recurrent LUTS due to residual or re-growing prostatic adenoma following prior Urolift surgery. It can be managed on a day-case basis with minimal surgical complications. However, the short follow-up period of three months limits our ability to assess the durability of symptom relief, retreatment rates, or long-term functional outcomes. Further prospective studies with extended follow-up and larger, more diverse cohorts are needed to fully establish the long-term role of Aquablation in this patient population.

## Data Availability

No datasets were generated or analysed during the current study.
